# *Rickettsia parkeri* and *Candidatus* Rickettsia andeanae in *Amblyomma maculatum* Group Ticks

**DOI:** 10.3201/eid2602.190664

**Published:** 2020-02

**Authors:** Bruce H. Noden, Megan A. Roselli, Scott R. Loss

**Affiliations:** Oklahoma State University, Stillwater, Oklahoma, USA

**Keywords:** Amblyomma maculatum, Rickettsia parkeri, Oklahoma, Candidatus Rickettsia andeanae, tickborne infections, vector-borne infections, United States, bacteria

## Abstract

We determined prevalence of *Rickettsia *spp. in 172 ticks of the *Amblyomma maculatum* group collected from 16 urban sites in Oklahoma City, Oklahoma, USA, during 2017 and 2018. Most ticks (59.3%) were collected from 1 site; 4 (2.3%) were infected with *Rickettsia parkeri* and 118 (68.6%) with *Candidatus* Rickettsia andeanae.

*Rickettsia parkeri*, part of the spotted fever group Rickettsia (SFGR), affects humans throughout much of the southern United States (*1*). Although *R. parkeri* in an engorged nymph was reported once in Oklahoma, *R. parkeri* has not been reported in adult *A. maculatum* ticks in Oklahoma or Kansas. To date, all test-positive adult ticks in Kansas and Oklahoma have been infected with *Candidatus* Rickettsia andeanae (*2*). The absence of *R. parkeri* in Oklahoma is surprising because it was detected in *A. maculatum* group ticks recovered from dogs in Arkansas counties bordering eastern Oklahoma (*3*) and in adult *A. maculatum* ticks in Texas (*4*), and *A. maculatum* ticks have been present in Oklahoma since the 1940s (*4*). We collected *A. maculatum* ticks in the Oklahoma City metropolitan area during May–August 2017 and 2018 and tested them for *Rickettsia* spp.

We selected 16 sites as part of a larger study of tickborne disease epidemiology (Figure). We performed collections during May–August by flagging vegetation or using CO_2_ traps (*5*). We completed identification by using established keys (*6*).

**Figure:**
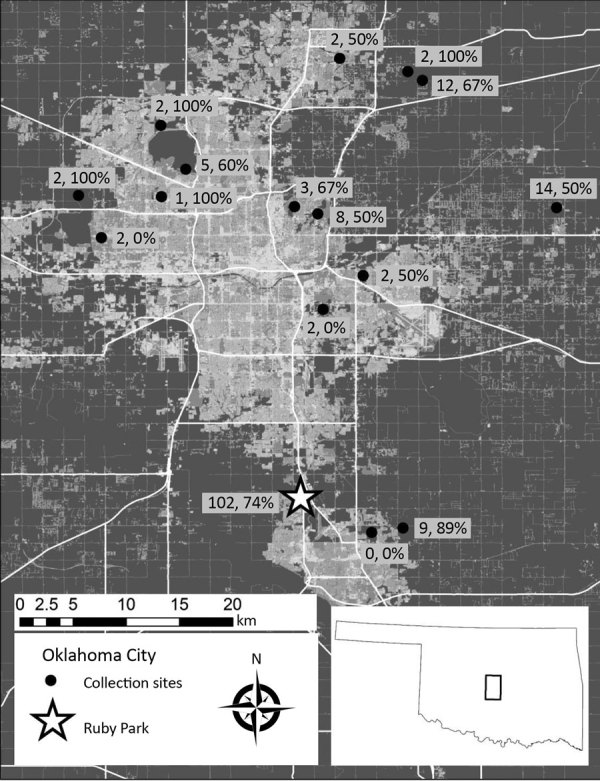
Locations where ticks of the *Amblyomma maculatum* group were collected (dots) in Oklahoma City, Oklahoma, USA. Numbers of *A. maculatum* ticks collected and percentage infected with *Candidatus* R. andeanae are indicated. Star indicates location where *Rickettsia parkeri*–infected ticks were collected. Figure constructed with ArcMap from highway data from the Environmental Systems Research Institute (Redlands, CA) and the US Geological Survey National Land Cover Database.

We tested field-collected ticks for rickettsial DNA by using established PCR protocols (*7*,*8*). To limit DNA contamination, we conducted DNA extractions by using site-specific reagents in a separate laboratory. After soaking adult ticks in deionized water for 30 minutes and surface-sterilizing with 70% ethanol, we longitudinally bisected ticks; we used one half for DNA extraction and stored the other half at −80°C. DNA extraction followed established protocols (*5*). In 2017, we screened all ticks by using assays targeting the *gltA* and *ompA* (*8*) genes and retested positive samples by using an assay targeting the *ompB* gene (primer pair 120–2788/120–3599) (*7*). In 2018, we initially screened ticks by using the *gltA* assay and confirmed the results with an *ompB* assay. 

We sequenced positive *ompB* amplicons bidirectionally by using an Applied Biosystems 3730 DNA Analyzer (https://www.thermofisher.com) at the Oklahoma State University Core Facility to identify bacterial species. We verified each resulting sequence by using BioEdit 7.2 (https://bioedit.software.informer.com) and aligned bidirectional sequences to create consensus sequences by using Clustal Omega (https://www.ebi.ac.uk/Tools/msa/clustalo). We compared resulting consensus sequences with GenBank submissions by using default conditions on BLAST (http://blast.ncbi.nlm.nih.gov/Blast.cgi), using the highest percentage sequence identity to determine species similarity.

We collected 172 adult ticks in the *A. maculatum* group (112 in 2017, 60 in 2018; 81 male [50 in 2017, 31 in 2018] and 91 female [62 in 2017, 29 in 2018]) from 15/16 sites across Oklahoma City (Figure). Most (59.3%) *A. maculatum* ticks were collected at 1 site in the southwestern metropolitan area consisting of grassland and deciduous shrubland and woodland surrounded by rapidly growing suburban developments (Figure). Most *A. maculatum* tick collections occurred in areas dominated by grassland with few woody plants and trees.

Initial screening of the 172 ticks detected 122 positive results, indicating a *Rickettsia* spp. prevalence of 70.9% (76.8% in 2017, 60.0% in 2018). Consensus sequences demonstrating 100% identity with the 850-bp portion of the *ompB* gene of *R. parkeri* Portsmouth (GenBank accession no. CP003341.1) and the 590-bp portion of the *ompA* gene of *R. parkeri* La Paloma (GenBank accession no. MG574938.1) were amplified from 4 (3.3%) positive *A. maculatum* ticks (3 males in 2017, 1 female in 2018). All 4 *R. parkeri*–infected ticks were from 1 site (Figure). The remaining 118 (96.7%) sequences from 122 amplicon-positive *A. maculatum* ticks demonstrated complete identity to homologous 850 bp portions of the *ompB* gene of *Candidatus* R. andeanae (GenBank accession no. GU395297.1). The overall *Candidatus* R. andeanae prevalence by sex was 72.8% for males (74% in 2017, 71% in 2018) and 64.8% for females (74.2% in 2017, 44.8% in 2018). Most *Candidatus* R. andeanae–infected ticks (74/118) were from the park with *R. parkeri*–positive ticks; however, *Candidatus* R. andeanae–positive ticks also were collected in 12 other sites (Figure). No dually infected ticks were identified.

We identified *A. maculatum* group ticks infected with *R. parkeri* and *Candidatus* R. andeanae in the Oklahoma City metropolitan area. Oklahoma lies at the western edge of 1 of the highest-incidence areas of SFGR in the United States (*1*). To date, no human rickettsiosis cases caused by *R. parkeri* have been reported in Oklahoma, possibly because of treatment based on nonspecific symptoms and the lifting of mandatory reporting to the Centers for Disease Control and Prevention (*9*). The low prevalence of *R. parkeri* in Oklahoma ticks differs from other areas of the United States, such as Virginia, where prevalence of *R. parkeri* is higher in *A. maculatum* ticks (*10*). *Candidatus* R. andeanae prevalence in *A. maculatum* ticks varies inversely with *R. parkeri* prevalence in some regions (*4*). Although *Candidatus* R. andeanae is not known to cause human illness (*4*), the high prevalence of *Candidatus* R. andeanae in Oklahoma ticks might interfere with *R. parkeri* development, limiting its distribution (*2*). The potential presence of this human pathogen in the largest metropolitan area in the state, and 1 of the largest in the central United States, necessitates thorough case evaluation of future SFGR cases in this region.
